# Thin-Film Solar Energy Absorber Structure for Window Coatings for Self-Sufficient Futuristic Buildings

**DOI:** 10.3390/mi14081628

**Published:** 2023-08-17

**Authors:** Haitham Alsaif, Jonas Muheki, Naim Ben Ali, Kaouther Ghachem, Jaymit Surve, Shobhit K. Patel

**Affiliations:** 1 Department of Electrical Engineering, College of Engineering, University of Ha’il, Ha’il City 81451, Saudi Arabia; 2 Department of Physics, Marwadi University, Rajkot 360003, Gujarat, India; 3 Department of Industrial Engineering, College of Engineering, University of Ha’il, Ha’il City 81451, Saudi Arabia; 4 Photovoltaic and Semiconductor Materials Laboratory, National Engineering School of Tunis, University of Tunis El Manar, Tunis 1002, Tunisia; 5 Industrial and Systems Engineering Department, College of Engineering, Princess Nourah bint Abdulrahman University, P.O. Box 84428, Riyadh 11671, Saudi Arabia; 6 Department of Electrical Engineering, Marwadi University, Rajkot 360003, Gujarat, India; 7 Department of Computer Engineering, Marwadi University, Rajkot 360003, Gujarat, India

**Keywords:** solar absorber, polarization insensitive, large-angle independent, energy efficient

## Abstract

Energy-efficient buildings are a new demand in the current era. In this paper, we present a novel metamaterial design aimed at achieving efficient solar energy absorption through a periodic MMA structure composed of a W-GaAs-W. The proposed structure can be implemented as the window coating and in turn it can absorb the incident solar energy and, then, this energy can be used to fulfill the energy demand of the building. Our results reveal significant improvements, achieving an average absorptance of 96.94% in the spectral range. Furthermore, we explore the influence of the angle of incidence on the absorber’s response, demonstrating its angle-insensitive behavior with high absorption levels (above 90%) for incidence angles up to 60° for TE polarization and 40° for TM polarization. The proposed structure presents a significant advancement in metamaterial-based solar energy absorption. By exploring the effects of structural parameters and incident angles, we have demonstrated the optimized version of our proposed absorber. The potential applications of this metamaterial absorber in self-sufficient futuristic building technologies and self-sustaining systems offer new opportunities for harnessing solar energy and are a valuable contribution to future developments in the fields of metamaterials and renewable energy.

## 1. Introduction

Metamaterials (MM) continue to attract researchers to intensively focus on their study [1] because of their vast applications, and electromagnetic properties [2], and metamaterial designs is quite achievable [3]. The aim of metamaterials is to provide effective properties of special materials not found in nature [4]. The intense study of metamaterials has provided new devices used in ultra-sensitive sensing [5], image processing [6], light controllers [7], surface-enhanced sensing [8], and perfect absorbers [9]. Perfect absorbers have sparked more attention in the research field because of their unique properties since their first demonstration as metamaterial perfect absorbers, with broader applications in organic solar cells [10], thermal imaging, etc. MM structures are commonly manifested as three-layered structures [11] following the metal–insulator–metal (MIM) structure [12]. The ground metal layer prevents the propagation of EM waves, the insulator layer provides coupling capacitance, and the resonator metal layer provides the impedance matching characteristics with free space [13]. Other kinds of layered structural designs have been engineered such as two-layer [14], four-layer [15], and multilayered structures. Research on how to attain broadband absorbers by trying different approaches such as adjusting the structures of the absorber, using geometrical shapes such as spherical structures, U-shaped structures, dumbbell-shaped structures, etc., continues to draw attention.

High optical loss materials, such as tungsten (W) [16], Ti [17], and others, can be used to successfully pick materials to achieve broadband absorptance. Utilizing TiN, Yu et al. were able to achieve an average absorption of more than 90% in the 360 nm to 1624 nm range [18]. In the range of 415.648–1597.39 nm, Li et al. used a double-size cross-shaped TiN absorber and found an absorption bandwidth of above 90% [19]. The utilization of Ti and related compounds has, therefore, been demonstrated by researchers due to its high melting point and superior optical loss. In a specific spectral range, high light absorption efficiency is possible [20]. Efforts are being made to study a wide absorption bandwidth using various shapes, sizes, and materials that have high efficiency, many applications, and are simple to fabricate. Some of these methods have demonstrated their ability to provide a wide spectrum of absorptance using metal nanostructures. The most traditional method is to simultaneously incorporate two or more distinct nano-resonators into a unit of metamaterial, which can broaden the structure’s absorption spectrum [21]. A pyramid-shaped solar absorber made of titanium carbide was published by Raza et al. [22] and demonstrated perfect absorption in the visible region as well as strong absorption of IR radiation up to 2000 nm with an efficiency of 88% in the visible range. MMA structures still pose huge disadvantages such as a narrow absorption bandwidth, and there is still scope for improving the absorption rate. An effective 99.87% absorbance at 239.35 THz was shown by a straightforward quad-band PPA built on all-metal nanostructures [23]. Improved second-harmonic production in the visible band (400–650 nm) was demonstrated using a nanostructured Au/ZnO multilayer [24]. An average absorption of 99.1% was observed under a typical incident THz wave using a six-band terahertz (THz) perfect metasurface absorber (PMSA) design [25]. A multilayer nanodisc-structured solar absorber and thermal emitter were made using W and TiN, with a dielectric layer of Si_3_N_4_. A greater than 90% bandwidth of 2929 nm was achieved [26]. Using transparent multilayer planar absorber coatings consisting of titanium (Ti) and iron dioxide (Fe_2_O_3_), buildings’ winter thermal management was improved [27]. There is still scope for a more efficient broadband solar absorber for certain applications. A similar design to that which we have proposed has been developed before by [28], and they achieved a broadband absorption from 200 nm to 1600 nm only, while with the same pyramid shape we have achieved broadband absorption till 2500 nm and for 870 nm bandwidth we achieved an absorption rate of more than 98%, which was 678 nm only. 

It is to be pointed out that very few researchers have focused on the application of solar absorbers as a window coating and the size aspect is also a major issue. So, here, we have reduced the size of a unit solar absorber cell and achieved a higher performance in terms of absorption, bandwidth, and change in absorption with respect to the incidence angle. We have also covered a unique application of solar absorbers in a very novel window coating. The predicted localized surface plasmon resonance (LSPR) [29] modes are created on top of the truncated pyramid MIM layered interface for transmission of incident electromagnetic waves through the suggested pyramidal-shaped absorber. This LSPR helps to make ultra-broadband absorption possible. When the LSPR modes couple to propagating surface plasmon resonance (PSPR) [30] modes at the interface of the GaAs layer and ground tungsten metallic layer [31], the absorption spectra in the metamaterial absorber are improved. 

We propose a broadband metamaterial absorber (MMA) based on an MIM structure with a thin pyramid-shaped resonator that can help to achieve a broadband, large-angle-independent absorption response in a range of solar spectral regions (UV, visible, and NIR). The proposed study is also compared with existing structures to improve the efficiency of the metamaterial absorber and attain more absorptance in the solar spectral regions in accordance with the air mass curve. Furthermore, the impact of geometrical parameters on the absorption is evaluated, taking into account that the light source is unpolarized and incident at an angle. Scanning (e-beam) lithography can be used to manufacture the suggested MMA truncated tungsten pyramid [32], and the GaAs substrate can be deposited using the low-pressure chemical vapor deposition (LPCVD) technique [33], and using the lithography process, the top pyramidical shape can be achieved. The proposed structure can be implemented as window coatings and in turn it can absorb the incident solar energy and, then, this energy can be used to fulfill the energy demand of the building. In this way, we can design energy self-sufficient buildings. Section 2 discusses the design procedure for the created MIM; Section 3 presents the findings in more detail; and Section 4 concludes this article. The proposed absorber will be useful for a variety of real-world applications because of the broad, large-angle-independent, polarization-sensitive, and stable absorption response that our simulation findings demonstrate.

## 2. Structure Design and Methods

**Figure 1:**
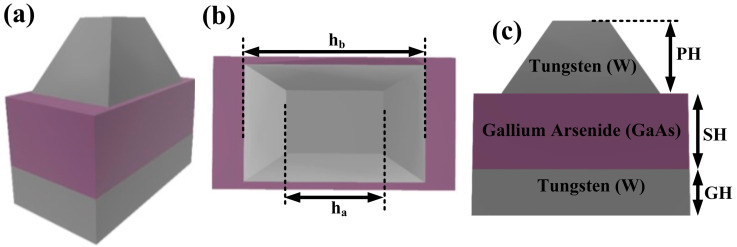
Schematic of the solar energy absorber structure that has been proposed: (**a**) 3D perspective, (**b**) top view, and (**c**) front view.

In Figure 1, the suggested MIM design is displayed. Tungsten (W) metal forms the top layer, which is a frustum pyramid, while GaAs serves as the intermediate layer’s insulator and W metal makes up the bottom layer, which is a square. We used the COMSOL multiphysics program to simulate the periodic metamaterial absorber (MMA) structure with suitable boundary conditions [34]. In this study, we propose a metamaterial design utilizing a periodic MMA structure to achieve efficient solar absorption. The design consists of a trilayer with tungsten (W) selected for the top and bottom layers, while a gallium arsenide (GaAs) layer serves as a semiconductor. GaAs was chosen due to its unique material properties such as a tunable refractive index, enabling us to tailor the optical properties and optimize the light absorption. Additionally, GaAs exhibits excellent thermal stability, ensuring the structure withstands heat generated during solar energy absorption without degrading its optical properties.

**Figure 2:**
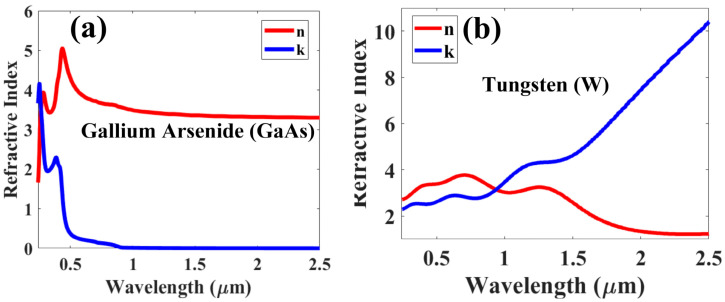
(**a**) GaAs’s refractive index plot; (**b**) tungsten’s (W) refractive index plot.

The simulations were performed using COMSOL, with the structure modeled as a periodic arrangement with periodic boundaries. Multiple unit cells were placed in a repeating pattern to achieve periodicity. A free tetrahedral meshing technique was employed to accurately capture the electromagnetic interactions. For the simulations, linearly polarized light with the electric field vector aligned along the x-axis was used as the light source, representing sunlight’s spectral distribution. The wavelength range from 200 nm to 2500 nm was utilized to assess the absorber’s performance across the UV, visible, and near-infrared portions of the spectrum. The substrate and ground films have thicknesses of GH and SH, respectively. The letters ha, hb, and PH stand for the top width, bottom width, and height of the frustum pyramid, respectively. With a refractive index of 1, air serves as the environment. Figure 2a,b show the graph of the refractive indices of tungsten (W) and gallium arsenide (GaAs) in the relevant band. The simulation’s geometric parameters were as follows: ha = 150 nm, hb = 185 nm, GH = 135 nm, SH = 300 nm, PH = 750 nm, and the periodicity of the unit cell is 200 nm. X-polarized light was incident, as is typical. The databases released by Raki et al. 1998 [35] and Majewski 1996 [36], respectively, served as the sources for the refractive indices of W and GaAs. The straightforward statement illustrating the link between absorption (A) and reflection can be used to compute the overall absorption of the metamaterial (R).

A + R + T = 1(1)

## 3. Results

### 3.1. Absorption Investigation under Solar Radiation

**Figure 3:**
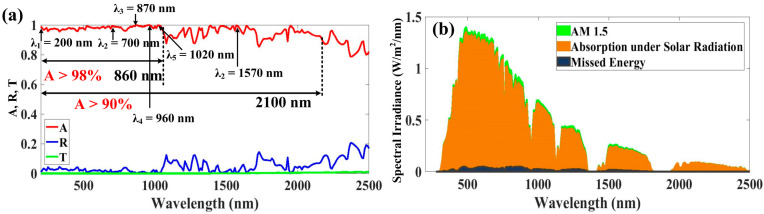
(**a**) Absorption, reflectance, and transmission of proposed structure. (**b**) Absorption of solar energy under AM 1.5, and missing solar energy for wavelengths of 200 to 2500 nm.

In Figure 2, which shows the absorption spectra of the suggested MMA, it can be seen that in the wavelength range of 200–1060 nm, transmission and reflection are almost zero and less than 0.05, respectively. The structure achieved an average absorption of 98.07% and an absorption of more than 98% up to 1060 nm. In the 200–2100 nm region, the simulation result depicted in Figure 3a likewise realized an absorption greater than 90%, with the average absorption of this band reaching 96.94%. At 200 nm, 700 nm, 870 nm, 960 nm, 1020 nm, and 1570 nm, near-perfect absorption peaks λ_1_, λ_2_, λ_3_, λ_4_, λ_5_, and λ_6_ are traced, with maximum absorptivity values of 99.03%, 99.52%, 99.95%, 99.96%, 99.81%, and 99.88%, respectively. In the band of interest, the average absorption values of the best design are as follows: 97.26% for UV, 98.03% for visible, and 93.95% for NIR, and the overall average absorption for the solar spectrum of 96.94% is also observed. Several findings show that the suggested improved absorber performs superbly, and the broadband absorption attained is the result of the conjunction of these almost perfect absorption peaks.

AM 1.5 equation can be used to calculate the incident solar energy [37]:

(2) $$\eta =\frac{\int_{\lambda_{min}}^{\lambda_{max}}\left(1-R\left(\lambda \right)\right)\cdot I_{\mathrm{AM}1.5}\left(\lambda \right)\cdot d\lambda }{\int_{\lambda_{min}}^{\lambda_{max}}I_{\mathrm{AM}1.5}\left(\lambda \right)\cdot d\lambda }$$

where *R* is the reflectance, *I*_AM1.5_ is the direct solar irradiance standard for an air mass of 1.5, and η is the normal solar absorptance. Here, $\lambda_{min}$ is 200 nm and $\lambda_{max}$ is 2500 nm. The AM 1.5 spectra are used to assess the absorptance response of the suggested solar energy absorber under irradiation, as illustrated in Figure 3b. The proposed absorber’s solar radiation and missed solar energy absorption are shown in Figure 3b, the green line displays the AM 1.5. The UV to NIR spectrum of the solar energy absorbed is given a lot of attention in the suggested MMA. In the 200 nm to 2100 nm range, we obtain a high absorption rate, which increases the capacity of our suggested absorber to absorb solar energy. Only a very tiny quantity is missing in this range of interest, considerably noticed in the visible region, as can be seen in Figure 3b.

### 3.2. Effect and Role of Each Layer in Absorbing Solar Energy

The proposed structure is a metal–insulator–metal (MIM) structure and each layer has its significant importance. The top metal layer provides the metamaterial properties due to its shape and, in return, we achieve the negative refractive index which stops the reflection of solar energy. The middle insulator layer of GaAs is used as a loss element and contributes the most to the absorption of solar energy. While the bottom metal layer of W is kept there for preventing the transmittance of solar energy.

**Figure 4:**
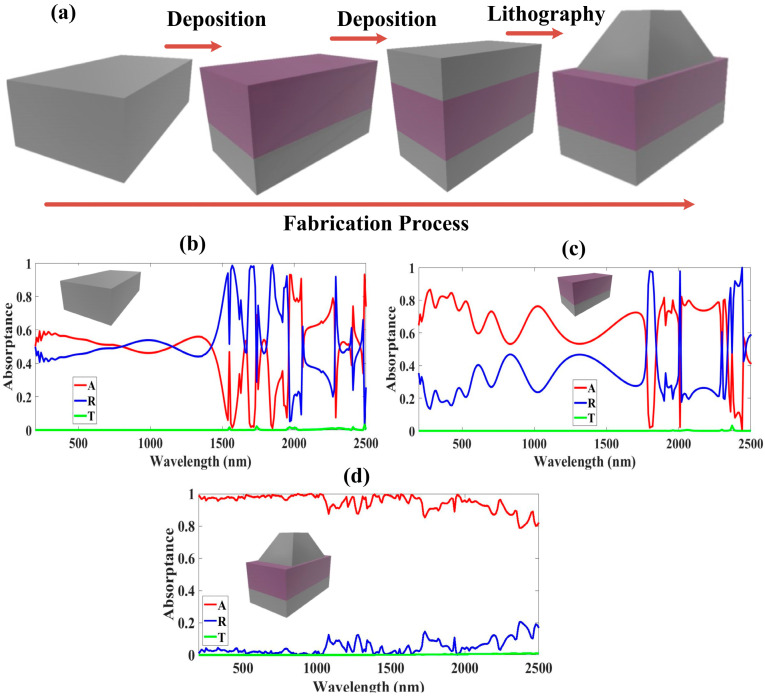
(**a**) A breakdown of the manufacturing of the proposed structure’s optimized truncated pyramid. (**b**) The ground layer’s corresponding absorptance response. (**c**) Absorptance response for substrate and ground layers. (**d**) The 3D plot’s absorptance reaction.

In Figure 4a, we indicate the necessary fabrication procedures of the proposed design, which consists of two simple processes of thin-film depositing and photolithography to achieve the pyramidal shape. From Figure 4b–d, we achieve a transmission of almost zero, T = 0, this implies Equation (1) can be written for absorptivity as A = 1 − R. The spectral absorption plots indicate a gradual increase in the absorption response. Looking at Figure 4b, we account for only the response of using the tungsten ground layer in an optimized state and we achieve average absorption in the UV, visible, and NIR of 56.56%, 53.64%, and 46.85%, respectively, and an overall average absorption of 48.58% and maximum absorptivity of 93.26%. In the following step, we added a substrate layer of GaAs to the previous tungsten ground layer, and its corresponding absorption plot is shown in Figure 4c, it indicates a maximum absorptivity of 86.52% and a corresponding overall average absorption of 63.81%. The absorption average in the UV, visible, and NIR is as follows: 78.61%, 72.38%, and 60.77%. Without the resonator, the absorption response is still oscillatory and overall averages are unstable and drastically changing, therefore, we access a combination of all three layers to achieve a near-perfect absorber, attaining maximum peaks of over 99% absorptance, as shown in Figure 3a. In Figure 4d, the simulation results are shown, and it is clear that in the 200–2100 nm wavelength range, there is almost no transmission and very little reflection. This suggests that the structure might achieve absorption of more than 90% in this range (bandwidth 1900 nm), whereas the average absorption of this band might be as high as 98%. The simulated absorber performs superbly, as shown by these results.

### 3.3. Structural Parameters’ Effects on the Absorption Spectra of the Proposed Design

**Figure 5:**
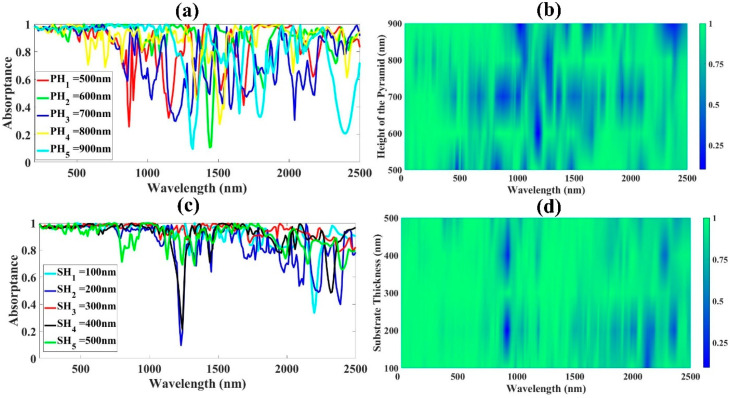
Structural characteristics affecting the performance of the absorption. (**a**) The impact of an incrase in the pyramid’s height on absorptance. (**b**) A color plot illustrating the impact of an incrase in the pyramid’s height (PH) on the proposed structure’s absorptance spectrum. (**c**) The effect of increasing the GaAs material-based substrate thickness, or SH, on the structure’s spectral absorptance, and (**d**) a Fermi plot showing how the thickness of the GaAs-based substrate (SH) affects the structure’s absorptance response.

A performance analysis of different parameters used in the proposed design was carried out to analyze the effect of each parameter (h_a_, h_b_, SH, PH) on the absorption. We have investigated this by changing the thickness of the substrate and resonator. Though there are no obvious effects to be outlined, we have discussed the details in terms of the absorptance rates for each thickness value of the substrate and resonator and other parameters. We have in the process identified the best parameter value for each parameter to optimize the structure to achieve a higher absorptance rate. Each parameter was adjusted one at a time by applying the appropriate parametric sweep condition during the process of simulation. Fixing SH, h_a_, and h_b_, we varied the pyramid height (PH) from 500 to 900 nm. Figure 5a, shows the absorption variations as the wavelength significantly increases. In a scenario where PH = 0, the proposed design will be composed of two square based ground metal layers and a thin substrate layer, and these two tend to present a weak absorptance, hence the need to analyze the effect of increasing the height of the pyramid (PH) in the design. The PH variation shows an average absorptance of >96% in the UV area, with the highest absorption of 97.06% in the visible region at 700 nm. The performance of the PH variation in the near-infrared region goes on increasing and dropping in a repetitive fashion, however, a maximum of 93.62% is also obtained in this region. The color plot in Figure 5b indicates a high concentration for a PH between 500 and 900 nm within the UV to visible spectrum. For the purposes of manufacturing, PH = 750 nm was considered in this simulation.

**Figure 6:**
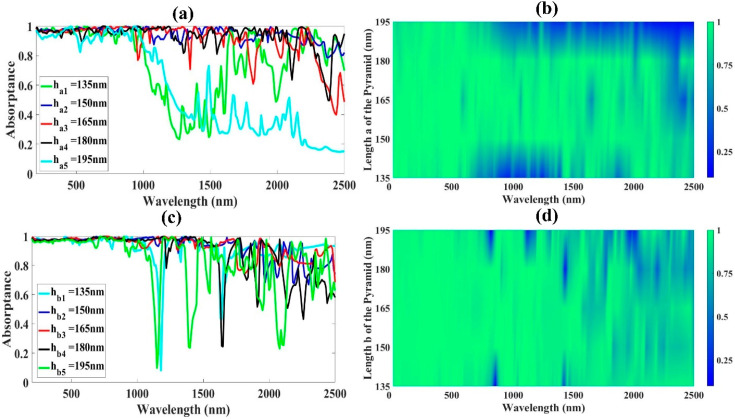
Structural characteristics affecting how the structure absorbs energy. In particular, (**a**) shows how lengthening the pyramid’s base affects how well the proposed structure absorbs energy. (**b**) A color plot illustrating the impact of lengthening the pyramid’s a side (h_a_) on the spectral absorptance response; (**c**) the effect of lengthening the pyramid’s b side (h_b_) on the proposed structure’s absorptance; and (**d**) a Fermi plot illustrating the impact of the pyramid’s hb side (based on tungsten) on the absorptance response.

While keeping other parameters constant, we added a thin insulator GaAs substrate layer (SH) to the structure in order to achieve broadband absorption. As shown in Figure 5c,d, this layer’s response exhibits a wide, nearly perfect absorptance, with the highest value of absorptance in the UV region of 97.36%. However, as the thickness increases, the intensity of the absorptance in this range decreases. The absorptance in the visible region rises to an average value of 98.03% as the thickness approaches 300 nm and then slightly declines to 96.03% as the thickness continues to rise. The NIR region’s activity is erratic, with intensities varying as the thickness increases. The average absorptance response is inefficient at both 135 nm and 190 nm, recording an average of 77.57% and 57.59%, respectively, as illustrated in Figure 6a,b, when the length ha is extended from 135 nm to 195 nm. The structural absorption response clearly drops as it reaches the band of strong interest beyond 180 nm, with the highest overall performance of 94.75% at 150 nm. The absorption performance of the structure is above 90% when h_a_ is between 150 and 180 nm (NIR region). In the next section, we examine the effect of parameter h_b_, the length b of the truncated pyramid as illustrated in Figure 6c,d. In the 185–195 nm range, the absorption band narrows. Since a spectral absorptance exceeding 90% can be attained, and because it is simple to construct the pyramid structure, we used h_b_ up to 185 nm in our proposed structure. The predetermined length ensures that the suggested structure will absorb energy efficiently by leaving a gap between the adjusted pyramids during the design and layering of the various cells.

### 3.4. Performance Effects of the Angle of Incidence

**Figure 7:**
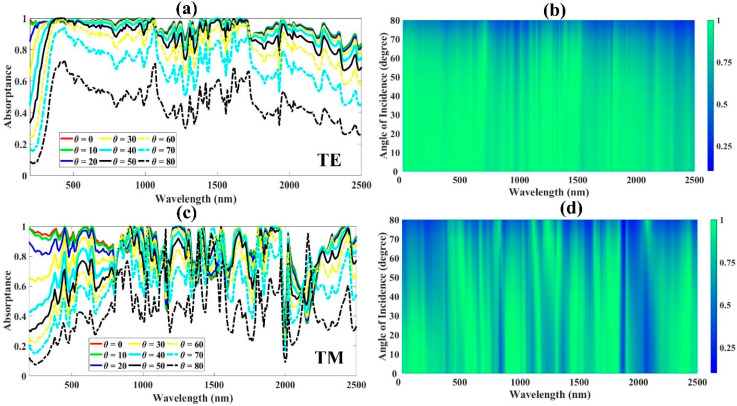
(**a**,**c**) Variations in the absorption when the incident angle is changed from 0° to 80° under polarized light, and (**b**,**d**) Fermi plots that show how the developed truncated-pyramid solar energy absorber responds to absorptance for different angles of incidence for TE and TM polarization modes.

The effect of the incidence angle on the reaction to absorption is depicted in Figure 7a. The TE polarization mode is used for the color plot at different angles of incidence displayed in Figure 7b. The created absorber offers an effective response from 0° to 60°. An undulating spectrum of absorption is visible at incidence angles of 70° and 80°. The proposed structure absorbs less in the UV, more in the visible, and above 80.97% for angles between 0° and 60° in the near-infrared, according to a shift in the absorption spectrum away from the ultraviolet area towards the visible and near-infrared. For the measured spectra (200–2500 nm), the absorption between 0° and 60° clearly shows the highest absorptance. In the UV region, we obtained average absorptances of 97.24%, 97.18%, 95.84%, 91.31%, 83.51%, and 73.66% between 0° and 60°. The overall average absorptances reached from the UV to the NIR in the range of (0–60°) are 95.08%, 94.99%, 94.56%, 93.45%, 91.29%, 87.51%, and 80.88%.

The absorption spectrum results in Figure 7c,d show that the absorption is minimal as the wavelength increases, even for relatively large angles of incidence between 40° and 80°. The resonance of incident light is improved by the modest oscillations at short wavelengths. It can be seen that the proposed absorber is, to some extent, insensitive to the incident angle. This makes the absorber useful for practical applications since it achieves polarization-sensitive absorption and large-angle-independent response, even though the TE and TM plots show different responses and broadband absorption above 90% in both cases. As we can observe, the absorptance is highly dependent on the angle of incidence in the short-wavelength range and this depends on the resonator geometry and angle of incidence, as we can observe in the figure. It may be that the pyramid shape is affecting the absorption in the short-wavelength region. 

### 3.5. Influence of Electric Field Distribution at the Six Perfect Peaks in XY and XZ Planes

**Figure 8:**
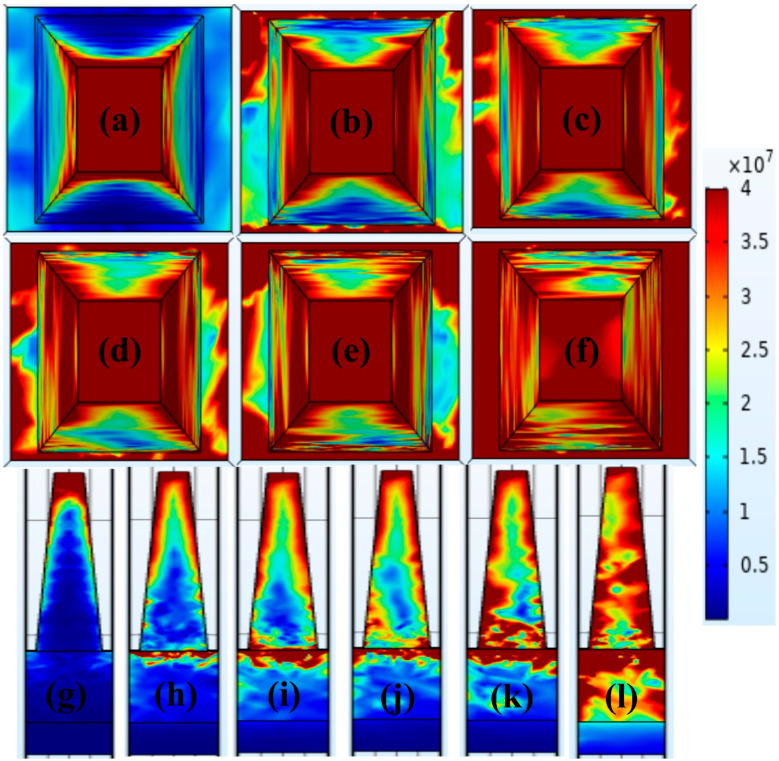
The electric field in the XY and XZ distributions of the truncated-pyramid-shaped solar absorber at peaks of 200 nm (**a**,**g**), 700 nm (**b**,**h**), 870 nm (**c**,**i**), 960 nm (**d**,**j**), 1020 nm (**e**,**k**), and 1570 nm (**f**,**l**), respectively.

As the wavelength increases from UV to NIR wavelengths, it is evident from the electric field distribution figures in Figure 8 that the electric field concentration is primarily dispersed at the top of the resonator of the proposed absorber and continually spreads to the insulator. The electric field is dispersed at the top of the truncated pyramid as shown in Figure 8a,g at the first absorption peak (λ_1_ = 200 nm), which is in the short-wavelength spectrum. As the electric fields propagate to the visible area at the second peak, λ_2_ = 700 nm, their concentration is distributed to the resonator top as well as at the interface of the GaAs layer and W film, as seen in Figure 8b,h. Electric fields are increasingly absorbed in the insulator layer at peaks at λ= 870 nm, 960 nm, and 1020 nm, as depicted in Figure 8c–e,j–k. At λ_6_ = 1570 nm, the middle layer exhibits a substantial increase in the electric field concentration, as seen in Figure 8f,l. The argument that insulator layers can greatly improve absorption is supported by the elevated electric field concentration in the insulator layer. Due to the coupling effects of the GaAs insulator layer with other metals, there is substantial absorption in the suggested configuration [34]. The achieved broadband absorption is the result of various contributing factors. The short-wavelength absorption is caused by LSPR on top of the truncated pyramid; the structural characteristics of the structure, the resonance cavity, and the bottom layer of the absorber are suitable for transmitting incident radiation. When the proposed structure is compared to the studies that are currently accessible, the proposed structure performs better, as shown in Table 1. As presented in the table, the proposed structure has a different response for TE and TM polarization and the angle dependency is also high after 40 degrees, which can be considered as the limitation of the study and this can be avoided by adjusting the window coatings to avoid these angles. 

## 4. Conclusions

In the UV, visible, and NIR wavelength ranges, we have created a pyramidal resonator-based metamaterial structure based on a regularly ordered metal–insulator–metal absorber structure. In a broad band of 860 nm from 200 to 1060 nm, the absorber achieves almost complete absorption, with an average absorption of 98.07%, and above 90% for a bandwidth of 2100 nm. Strong interaction between the metal and the insulator increases this broadband absorption. At angles up to 60° for the TE mode and 40° for the TM mode, absorption levels exceeding 90% are maintained. The suggested and designed absorber structure is polarization-dependent and insensitive at high incident angles. The developed solar energy absorber’s absorption performance is considerably enhanced by the resonator (a truncated pyramid) and substrate (an insulator layer). In order to achieve large-scale practical uses of MMA, such as energy harvesting and filters, the developed absorber may offer further breakthroughs and developments. The proposed structure can be implemented as window coatings and in turn it can absorb the incident solar energy and, then, this energy can be used to fulfill the energy demand of the building. In this way, we can design energy self-sufficient buildings.

## Figures and Tables

**Table 1:** Comparison of the created MMA with other studies.

Model	Interval	Average % of Overall Absorption	Bandwidth (Absorption > 90%)	Bandwidth (Absorption > 98%)	Angle Insensitive
**Developed** **Pyramid MMA**	**200–2500 nm**	**96.94**	**2100 nm**	**860 nm**	**TE (0–60°)** **TM (0–40°)**
Ref. [38]	8.88–30 µm	96.57	9.56 µm	-	(0–60°)
Ref. [39]	400–1500 nm	96.64	300 nm	-	-
Ref. [40]	0.3–30 GHz	71.82	14 GHz	2 GHz	-
Ref. [28]	300–1600 nm	98	900 nm	678 nm	TE (0–40°) TM (0–20°)
Ref. [41]	100–1000 THz	99	160 THz	700 THz	(0–55°)
Ref. [42]	8–30 µm	92	4 µm	-	(0–60°)

## Data Availability

Data will be made available at a reasonable request to the corresponding author.
